# Predicting Infant Sleep Patterns From Postpartum Maternal Mental Health Measures: Machine Learning Approach

**DOI:** 10.2196/78937

**Published:** 2026-02-03

**Authors:** Rawan AlSaad, Raghad Burjaq, Majid AlAbdulla, Alaa Abd-alrazaq, Javaid Sheikh, Rajat Thomas

**Affiliations:** 1 Weill Cornell Medical College in Qatar, 2700 Education City, Doha, Qatar, 974 44928830; 2 Women's Wellness and Research Center, Hamad Medical Corporation, Doha, Qatar; 3 Mental Health Services, Hamad Medical Corporation, Doha, Qatar; 4 College of Medicine, Qatar University, Doha, Qatar

**Keywords:** artificial intelligence, sleep, postpartum, mental health, depression, women's health.

## Abstract

**Background:**

Postpartum maternal mental health (MMH) symptoms, including depression, anxiety, and childbirth-related post-traumatic stress disorder, are known to influence infant sleep trajectories. While previous research has examined their individual and combined associations, the predictive utility of these MMH symptoms for the early identification of infant sleep problems through machine learning (ML) remains understudied.

**Objective:**

This study aimed to examine whether postpartum MMH measures can predict infant sleep outcomes during the first year of life. The analysis focused on 2 clinically relevant sleep indicators: (1) nocturnal sleep duration and (2) night awakening frequency.

**Methods:**

A total of 409 mother-infant dyads were included in the study. Predictor variables comprised postpartum MMH symptoms assessed between 3 and 12 months postpartum, along with sociodemographic characteristics of mothers and infants. MMH symptoms were measured using 3 validated instruments: the Edinburgh Postnatal Depression Scale, the Hospital Anxiety and Depression Scale, and the City Birth Trauma Scale. Infant sleep outcomes were assessed using the Brief Infant Sleep Questionnaire. Six supervised ML algorithms were evaluated: logistic regression, random forest, support vector classifier, extreme gradient boosting, Light Gradient Boosting Machine, and multilayer perceptron. Post hoc feature importance analyses were conducted to identify the most influential predictors associated with each infant sleep outcome.

**Results:**

All models demonstrated high predictive performance. The best model achieved a precision-recall area under the curve of 0.92, *F*_1_-score of 0.84, and accuracy of 0.88 for predicting short nocturnal sleep duration. For frequent night awakenings, the top precision-recall area under the curve was 0.91, with an *F*_1_-score of 0.78 and accuracy of 0.85. Key predictors included maternal age and total scores from the Edinburgh Postnatal Depression Scale, Hospital Anxiety and Depression–Anxiety subscale, and City Birth Trauma Scale, with individual symptom items offering additional discriminative value.

**Conclusions:**

ML models can accurately predict which infants are at risk for suboptimal sleep based on MMH measures, enabling personalized, responsive, and developmentally informed postpartum care that promotes long-term maternal and infant well-being.

## Introduction

Infant sleep plays a foundational role in early neurodevelopment, with significant implications for cognitive functioning, emotional regulation, physical growth, and long-term health outcomes [1-3]. During the first year postpartum, infant sleep patterns are highly dynamic and marked by individual variability in both nocturnal sleep duration and the frequency of night awakenings. Insufficient or fragmented sleep during this critical period has been associated with impaired memory consolidation, behavioral dysregulation, and suboptimal emotional development [4,5].

A growing body of research [6-12] has demonstrated that maternal mental health (MMH) symptoms during the postpartum period, including depression [13,14], anxiety [8,15], and childbirth-related post-traumatic stress disorder (CB-PTSD) [16,17], are associated with alterations in infant sleep architecture. However, the underlying mechanisms through which these maternal conditions influence infant sleep remain poorly understood [18]. Furthermore, most prior studies have examined these symptoms in isolation or as covariates, without evaluating their collective predictive value using integrative modeling approaches.

In parallel, machine learning (ML) approaches offer a powerful alternative to traditional regression techniques. Beyond simply reproducing associations already established using regression or structural equation models, ML offers added value by flexibly capturing nonlinear relationships and higher-order interactions among MMH symptoms and covariates. In this context, ML models can benchmark a range of algorithms on predictive performance, support individual-level risk stratification, and highlight symptom patterns that are most informative for early identification of infants at risk of sleep disturbance. In turn, this can refine existing theoretical models and guide more targeted, data-driven clinical decision support.

Recent studies have demonstrated the utility of ML in various infant sleep and postpartum mental health applications [19]. For example, Wang et al [20] developed an automated sleep-stage classifier using heart rate and respiratory rate data to predict white matter development in preterm infants. Similarly, Werth et al [21] designed a deep learning–based system for sleep-stage classification in preterm infants using electrocardiogram (ECG) signals. Additionally, the Sleep Well Baby project introduced a real-time sleep-wake state prediction algorithm based on physiological signals, facilitating improved monitoring in neonatal intensive care units [22]. In another study, Chang et al [23] utilized a multimodal wearable device to collect audio, ECG, and motion data, employing transformer-based neural networks to classify infant sleep/wake states with high accuracy. Furthermore, Huang et al [24] applied ML models to classify and identify infant sleep positions. However, existing ML-based studies have mostly focused on characterizing infant sleep problems using demographic, behavioral, or sensor-derived features, without explicitly leveraging MMH symptoms as primary predictors. To date, no study has examined whether postpartum MMH symptoms can be used, in conjunction with ML methods, to predict infant sleep patterns during the first year of life.

**Figure 1.**
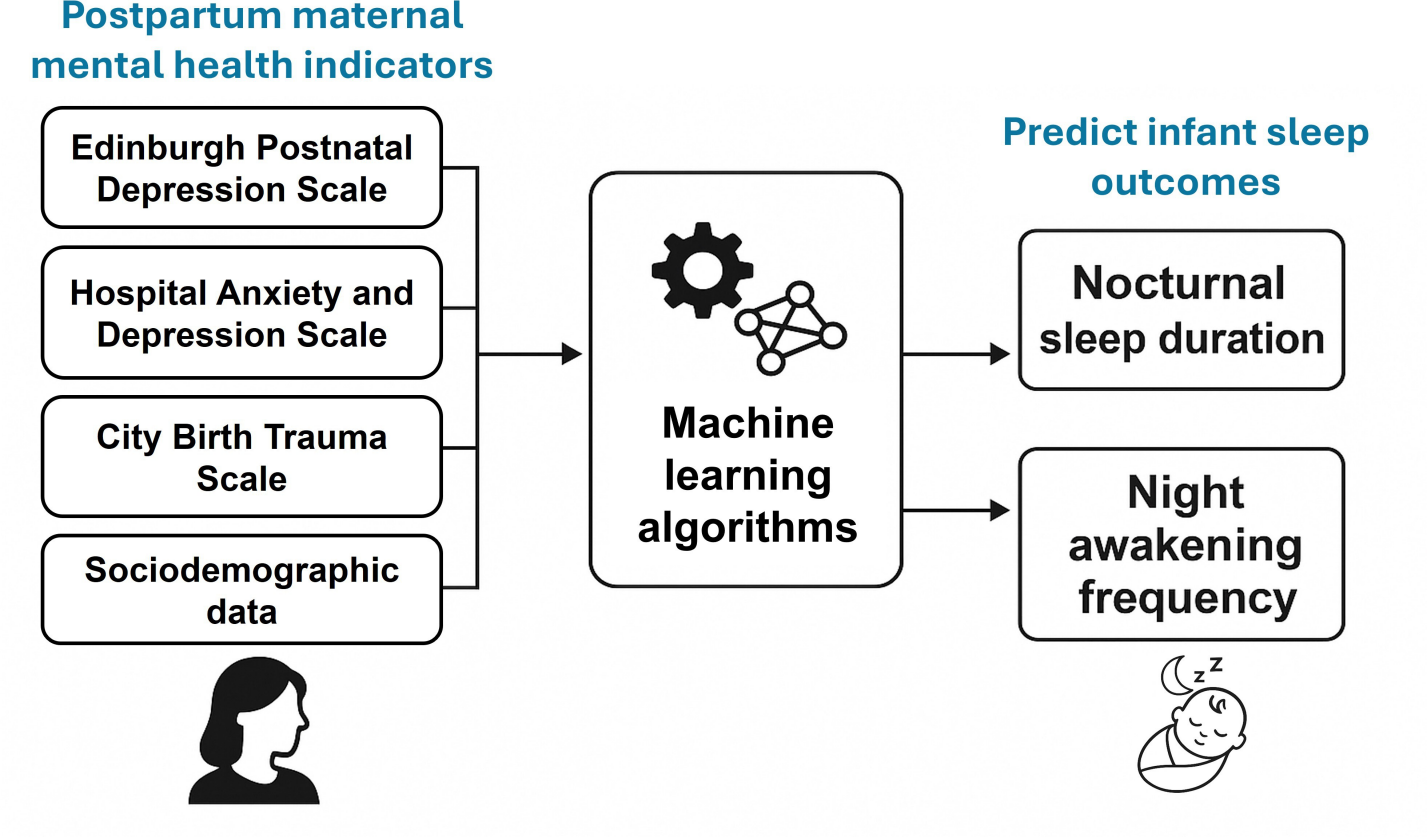
Overview of the machine learning framework used to predict infant sleep patterns based on postpartum maternal mental health indicators.

The present study addresses this gap by leveraging ML methods to predict infant sleep trajectories across the first year postpartum based on MMH symptoms and sociodemographic characteristics of mothers and infants. Specifically, we aimed to evaluate the performance of six supervised ML models in predicting two clinically relevant sleep outcomes: nocturnal sleep duration and the frequency of night awakenings (Figure 1). In addition, feature importance analyses were conducted to identify key MMH predictors associated with each outcome.

We hypothesized that postpartum MMH symptom measures, in combination with basic maternal-infant characteristics, would enable supervised ML models to accurately predict infant sleep outcomes. To operationalize this hypothesis, we addressed the following research questions: (1) Can MMH indicators and sociodemographic characteristics accurately predict infant sleep outcomes (ie, nocturnal sleep duration and night awakening frequency) during the first year postpartum using ML models? and (2) Which MMH features are most predictive of infant sleep outcomes across the first year postpartum? By characterizing the predictive utility of MMH symptoms and elucidating the most influential features, this study seeks to inform early screening and intervention strategies to optimize both MMH and infant developmental well-being.

## Methods

### Study Population and Data Sources

This study utilized a publicly available dataset [11] comprising 410 mother-infant dyads, collected via an online cross-sectional survey conducted between June and September 2020 at a university hospital in Switzerland. Eligible participants were birth mothers aged 18 years or older with infants between 3 and 12 months of age at the time of data collection and with no reported major neonatal complications.

The dataset included measures of MMH symptoms, infant sleep outcomes, and sociodemographic characteristics of both mothers and infants. A detailed description of the input features used in the analysis is provided in Multimedia Appendix 1. One mother-infant dyad was excluded due to missing information on nocturnal sleep duration, resulting in a final sample of 409 dyads.

### Data Elements

#### MMH Measures

MMH symptoms were assessed using 3 validated self-report instruments: the Edinburgh Postnatal Depression Scale (EPDS), the Hospital Anxiety and Depression Scale-Anxiety subscale (HADS-A), and the City Birth Trauma Scale (CBTS). These measures were selected to comprehensively capture postpartum symptoms of depression, anxiety, and CB-PTSD, respectively.

The EPDS is a 10-item screening tool designed to detect symptoms of postnatal depression in women [25]. It focuses on emotional and cognitive symptoms experienced during the preceding week, excluding somatic complaints that may overlap with normal postpartum changes. Each item is scored on a 4-point Likert scale, and total scores ranging from 0 to 40, with higher scores indicating greater symptom severity.

The HADS-A is the anxiety subscale of the Hospital Anxiety and Depression Scale [26]. It consists of 7 items that assess the frequency and severity of anxiety symptoms experienced during the preceding week. Responses are rated on a 4-point scale, yielding a total score ranging from 0 to 21, where higher scores reflect more severe anxiety symptomatology.

The CBTS is a 29-item instrument [27] specifically developed to assess CB-PTSD symptoms based on the Diagnostic and Statistical Manual of Mental Disorders, Fifth Edition (*DSM-5*). The scale is divided into 2 subscales: the birth-related symptoms subscale, which assesses intrusion and avoidance symptoms as well as a subset of negative mood items, and the general symptoms subscale, which captures remaining negative cognition and hyperarousal symptoms. The total score for the *DSM-5*–based items ranges from 0 to 60, with higher scores indicating greater severity of CB-PTSD symptoms. Together, these 3 instruments provided a multidimensional assessment of postpartum MMH, enabling the identification of symptom patterns relevant to infant sleep outcomes.

#### Infant Sleep Measures

Infant sleep was assessed using the Brief Infant Sleep Questionnaire (BISQ), a widely used and validated parent-report instrument designed to evaluate sleep behavior in infants and toddlers [28]. Mothers were asked to report on their infant’s sleep patterns over the preceding week, including total nocturnal sleep duration (between 7:00 PM and 7:00 AM), frequency of night awakenings, and method of falling asleep. For the purposes of this study, 2 primary sleep outcomes were derived and categorized as binary variables: nocturnal sleep duration and night awakenings, both of which serve as indicators of infant sleep quality.

Nocturnal sleep duration was classified as either normal (coded as 0) or insufficient (coded as 1). Infants were categorized as having normal nocturnal sleep if their reported sleep duration was ≥9 hours, for all infant age groups. Infants who slept for less than 9 hours per night were classified as having insufficient nocturnal sleep duration. This threshold aligns with prior research and pediatric sleep guidelines that recommend a minimum of 9 hours of nighttime sleep for infants aged 3 to 12 months [29] .

Night awakenings were categorized based on age-specific thresholds. For infants aged 3 to 6 months, normal was defined as ≤3 awakenings per night. For infants aged 6 to 9 and 9 to 12 months, normal was defined as ≤2 awakenings per night. Infants exceeding these thresholds were classified as having frequent night awakenings, consistent with existing sleep research indicating that night waking typically decreases with age as self-regulation improves [30].

Nocturnal sleep duration and night-awakening frequency were modeled as separate primary outcomes because they capture distinct dimensions of infant sleep—quantity (duration) versus continuity (awakenings)—that may have partially different determinants (eg, circadian scheduling/feeding patterns versus arousal regulation) and lead to different clinical actions. Their measurement properties also differ (duration: continuous; awakenings: count/ordinal), warranting distinct modeling approaches and metrics. Analyzing them separately preserves interpretability of feature effects and supports symptom-targeted guidance. Although the 2 domains can co-occur, our predictive focus is outcome-specific.

### Data Preprocessing

Preprocessing steps included computing total scores for the maternal mental health instruments (EPDS, HADS-A, and CBTS) according to their manuals and recoding response options for consistency across instruments. For each instrument, both the individual item responses and the derived total scores were retained as candidate predictors, allowing the models to leverage overall symptom burden as well as more fine-grained symptom patterns (eg, specific anxiety, depression, or trauma-related items). Missing values were imputed (numerical features: mean; categorical features: mode). Numerical features were then standardized (*z* score), and categorical features were one-hot encoded to ensure consistent transformations during model training and evaluation. This procedure yielded a clean, model-ready feature matrix for all classifiers. Because the study’s primary aim was to evaluate predictive performance rather than coefficient-level inference, we did not perform formal multicollinearity diagnostics (eg, VIF). Including both total scores and item-level responses intentionally introduces some correlation among predictors; however, many of the algorithms employed (eg, tree-based learners and regularized linear models) are designed to handle correlated and partially redundant features by down-weighting or shrinking less informative variables. Potential overfitting from the expanded feature space was further mitigated through cross-validated hyperparameter tuning and evaluation on a held-out test set.

### ML Models

Six ML algorithms were employed to predict infant sleep outcomes based on MMH measures and demographic features. These models were selected to represent a diverse range of linear and nonlinear classifiers, including both ensemble and neural network-based approaches.

- Logistic regression: A linear classification algorithm that estimates the probability of a binary outcome based on a weighted combination of input features.
- Random forest: An ensemble learning method that constructs multiple decision trees during training and outputs the class that is the mode of the predictions of the individual trees.
- Support vector classifier: A kernel-based method that identifies the optimal hyperplane separating classes in a high-dimensional feature space.
- extreme gradient boosting (XGBoost): A gradient-boosted decision tree algorithm known for its scalability and performance. It builds an ensemble of weak learners sequentially, optimizing residual errors from prior iterations.
- Light gradient boosting machine (LightGBM): A gradient boosting framework that uses histogram-based learning and leaf-wise tree growth.
- Multilayer perceptron (MLP): A feedforward artificial neural network composed of fully connected layers. It captures complex, nonlinear interactions among features and is trained using backpropagation.

Each questionnaire item and each derived total score was treated as a separate candidate predictor. Modern supervised ML algorithms (eg, tree-based ensembles and regularized models) are generally robust to moderately correlated predictors and can down-weight or ignore redundant features during training, so including both item-level and total-score features does not compromise model learning or model behavior; instead, it allows the algorithm to “decide” whether predictive signal is better captured at the composite-score or item level.

### Models Training and Evaluation Strategy

Both outcome variables exhibited class imbalance. For nocturnal sleep duration, 359/409 infants (87.8%) were classified as normal (class 0) and 50/409 (12.2%) as insufficient (class 1). For night awakenings, 346/409 infants (84.6%) were classified as normal (class 0) and 63/409 (15.4%) as elevated (class 1). To address this, we applied 2 strategies. First, we evaluated each model using 4 sampling methods: no sampling, random upsampling, random downsampling, and synthetic minority oversampling technique (SMOTE). This allowed us to assess the impact of different data distributions on model performance. Second, we used evaluation metrics suited for imbalanced data. In addition to accuracy, we computed the precision-recall area under the curve (PR-AUC), which focuses on the minority class and is not influenced by the number of true negatives. We also reported the *F*_1_-score, the harmonic mean of precision and recall, which balances false positives and false negatives. Together, these strategies ensured reliable evaluation of model performance in the context of class imbalance.

All analyses were conducted at the level of the mother-infant dyad. The dataset (N=409 dyads) was randomly split into training (327/409, 80%) and test (82/409, 20%) sets, using stratified sampling to preserve the proportion of infants with nocturnal sleep disturbance and frequent night awakenings in both partitions. All model development (including hyperparameter tuning and internal validation) was performed exclusively on the training set. All analyses were implemented in Python and performed on a high-performance computing node equipped with an NVIDIA A100 GPU (80 GB memory).

To quantify the variability and robustness of model performance, we additionally performed stratified 5-fold cross-validation within the training set for each model-sampling combination. For every fold, we computed PR-AUC, accuracy, and *F*_1_-score and summarized their distribution across folds. Final performance for each model was then evaluated on the held-out test set.

### Models Explainability

To characterize which MMH and covariate features contributed most to predictions, we first computed model-based feature importance for the best-performing model for each outcome (as determined by PR-AUC on the held-out test set), using the model’s native importance measure. To further enhance interpretability, we then performed a post hoc explainability analysis using Shapley additive explanations (SHAP). For each outcome, we computed SHAP values for all input features. SHAP values quantify the marginal contribution of each feature to the predicted probability of the positive (sleep disturbance) class for individual mother-infant dyads. We summarized global importance by the mean absolute SHAP value across participants and visualized the distribution of feature effects using SHAP summary (beeswarm) plots, as complementary views to the main feature-importance analyses.

### Ethical Considerations

This study did not involve the collection or generation of original human subject data. Instead, it utilized publicly available, deidentified data from a licensed source. As such, institutional review board approval and informed consent were not required.

## Results

### Participant Characteristics

A total of 409 mother-infant dyads were included. Participant characteristics and summary measures are shown in Table 1. The mean maternal age was 30.20 (SD 4.36) years. Nearly half held a university degree (192/409, 46.9%); 388 out of 409 (94.9%) were in a couple relationship. Overall, 51.6% (211/409) of the infants were female and 48.4% (198/409) were male. The mean gestational age at birth was 39.11 (SD 1.90) weeks. At assessment, infants were distributed as follows: 147/409 (35.9%) were aged 3 to <6 months, 133/409 (32.5%) aged 6 to <9 months, and 129/409 (31.5%) aged 9 to <12 months. MMH means were 9.06 (SD 6.76) on the EPDS, 7.85 (SD 4.26) on the HADS-A, and 13.15 (SD 10.81) on the CBTS.

**Table 1.** Sample characteristics and key measures (N=409).

Domain and variable	Value
Maternal	
Age (y), mean (SD)	30.20 (4.36)
Education, n (%)	
University degree	192 (46.9)
Applied Science/Tech diploma	88 (21.5)
Postsecondary/apprenticeship	103 (25.2)
Completed compulsory school	24 (5.9)
No formal education	2 (0.5)
Marital status, n (%)	
Couple relationship	388 (94.9)
Single	14 (3.4)
Separated/divorced/widowed	7 (1.7)
Pregnancy/birth	
Gestational age at birth (wk), mean (SD)	39.11 (1.90)
Infant	
Sex, n (%)	
Female	211 (51.6)
Male	198 (48.4)
Age group, n (%)	
3 to <6 mo	147 (35.9)
6 to <9 mo	133 (32.5)
9 to <12 mo	129 (31.5)
Maternal mental health, mean (SD)	
EPDS^a^ total	9.06 (6.76)
HADS-A^b^ total	7.85 (4.26)
CBTS^c^ total	13.15 (10.81)

a EPDS: Edinburgh Postnatal Depression Scale.

b HADS-A: Hospital Anxiety and Depression Scale-Anxiety Subscale.

c CBTS: City Birth Trauma Scale.

### Prediction of Nocturnal Sleep Duration

#### Models Performance

**Figure 2.**
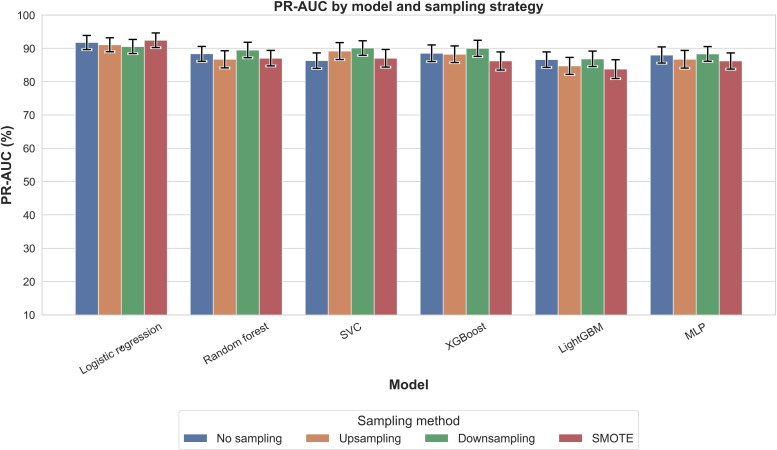
Comparison of the precision-recall area under the curve (PR-AUC) across models and sampling methods for outcome nocturnal sleep duration. PR-AUC quantifies how well a model can distinguish positive cases (infants with insufficient nocturnal sleep duration) from negative ones across various thresholds, especially under class imbalance conditions. LightGBM: light gradient boosting machine; MLP: multilayer perceptron; SMOTE: synthetic minority oversampling technique; SVC: support vector classifier; XGBoost: extreme gradient boosting.

**Figure 3.**
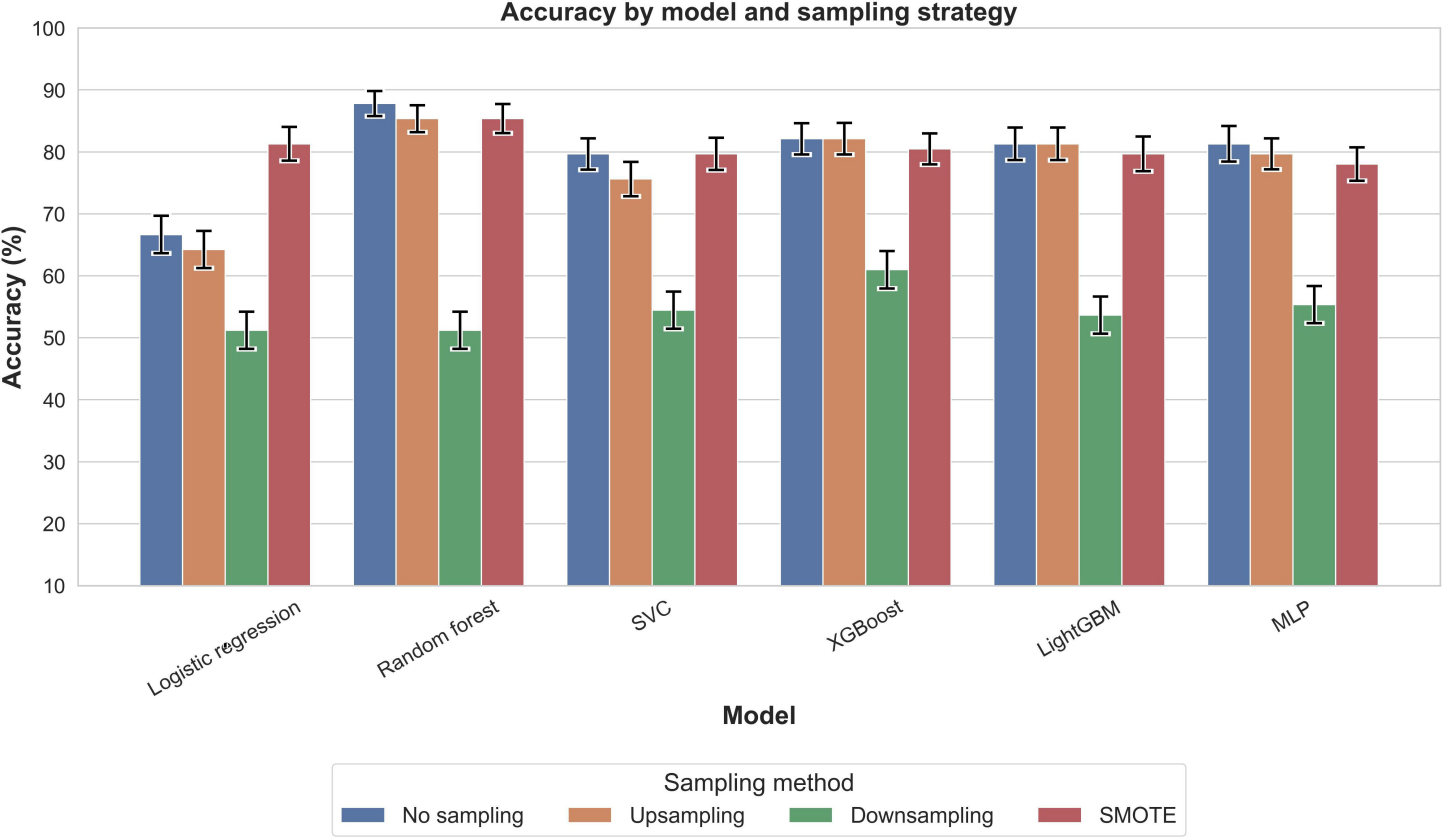
Comparison of accuracy across models and sampling methods for outcome nocturnal sleep duration. Accuracy represents the overall proportion of correct predictions, combining both positive and negative cases, and provides a broad measure of model correctness. LightGBM: light gradient boosting machine; MLP: multilayer perceptron; SMOTE: synthetic minority oversampling technique; SVC: support vector classifier; XGBoost: extreme gradient boosting.

**Figure 4.**
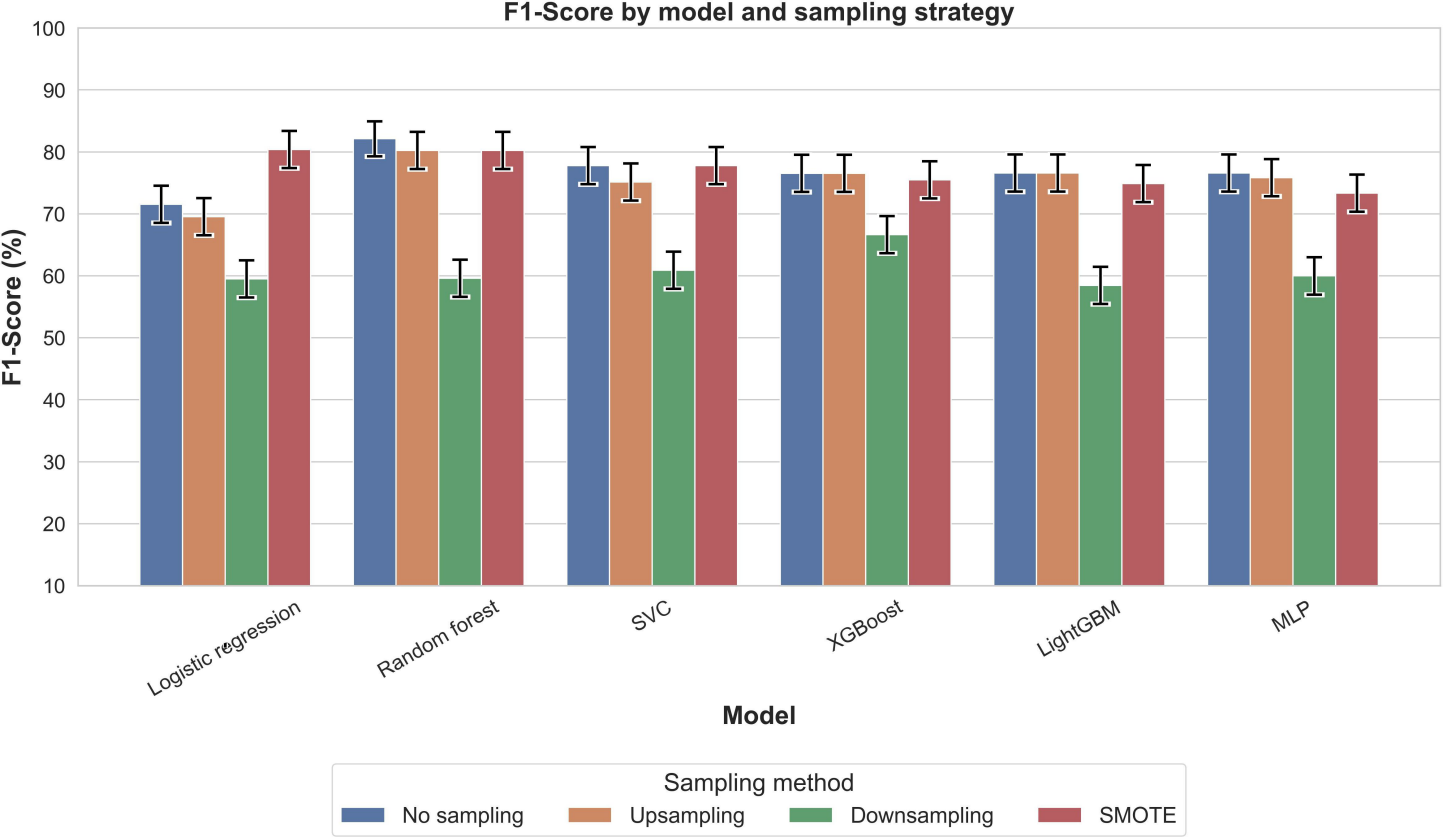
Comparison of *F*_1_-score across models and sampling methods for outcome nocturnal sleep duration. The *F*_1_-score balances precision and recall, making it a valuable metric for assessing model performance in the context of imbalanced datasets. LightGBM: light gradient boosting machine; MLP: multilayer perceptron; SMOTE: synthetic minority oversampling technique; SVC: support vector classifier; XGBoost: extreme gradient boosting.

Figure 2 presents PR-AUC values for each model across 4 sampling strategies. All configurations achieved PR-AUC values above 0.88, with XGBoost with SMOTE highest (0.931), followed by logistic regression with SMOTE (0.924). Accuracy (Figure 3) showed greater variability, with XGBoost without sampling highest (0.886), followed by random forest without sampling or with upsampling (0.878). *F*_1_-scores (Figure 4) mirrored these trends: XGBoost without sampling achieved the highest *F*_1_ (0.840), followed by random forest (0.821) and support vector classifier/MLP (0.820) with either no sampling or SMOTE.

#### Feature Importance Analysis

**Figure 5.**
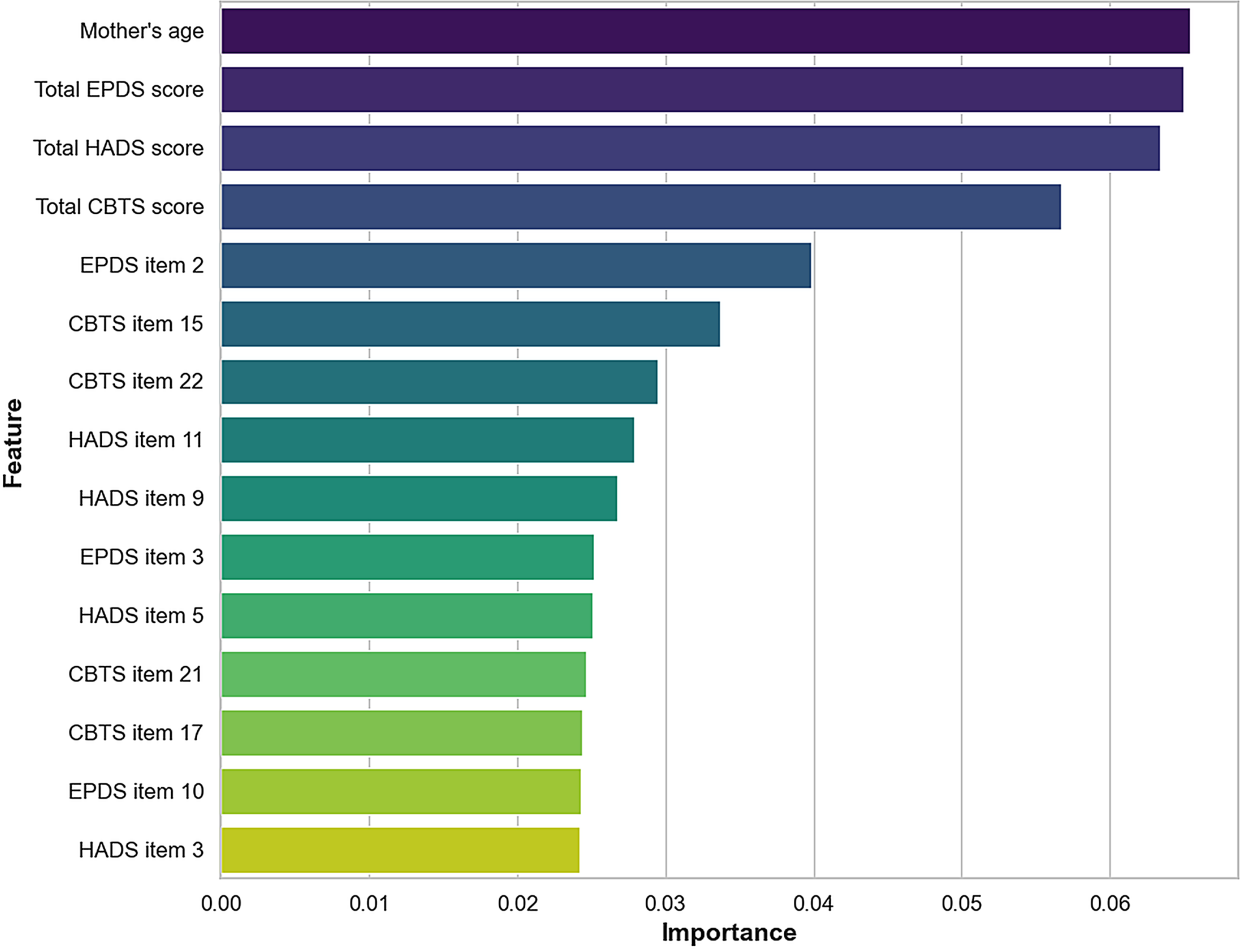
Feature importance analysis for outcome nocturnal sleep duration. EPDS: Edinburgh Postnatal Depression Scale; HADS: Hospital Anxiety and Depression Scale; CBTS: City Birth Trauma Scale.

Figure 5 shows the most influential predictors of short nocturnal sleep duration: maternal age and total scores on the EPDS, HADS-A, and CBTS. Individual items also contributed meaningfully, particularly EPDS Item 2 (I have looked forward with enjoyment to things) and CBTS Item 15 (Feeling detached from others). To further probe how individual feature values contributed to predictions for the best-performing model, we examined SHAP summary plots for the nocturnal sleep outcome (Multimedia Appendix 1). These global SHAP patterns were broadly consistent with the main feature-importance rankings and illustrate how higher maternal symptom scores tend to shift predictions toward increased risk of nocturnal sleep disturbance.

### Prediction of Frequent Night Awakenings

#### Models Performance

**Figure 6.**
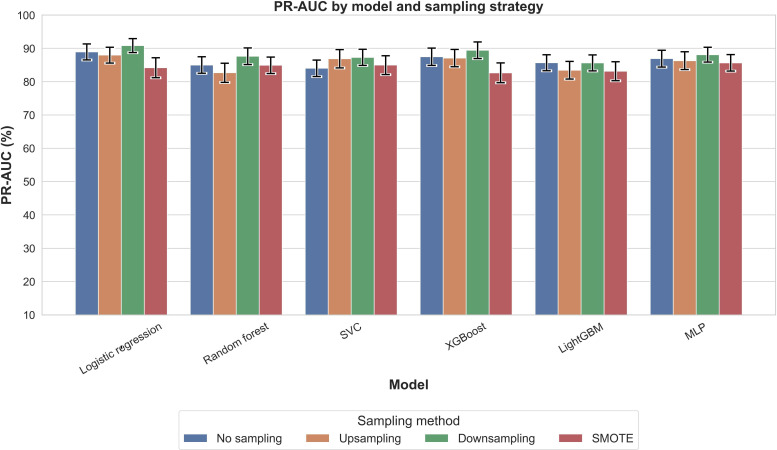
Comparison of the precision-recall area under the curve (PR-AUC) across models and sampling methods for outcome night awakenings frequency. LightGBM: light gradient boosting machine; MLP: multilayer perceptron; SMOTE: synthetic minority oversampling technique; SVC: support vector classifier; XGBoost: extreme gradient boosting.

**Figure 7.**
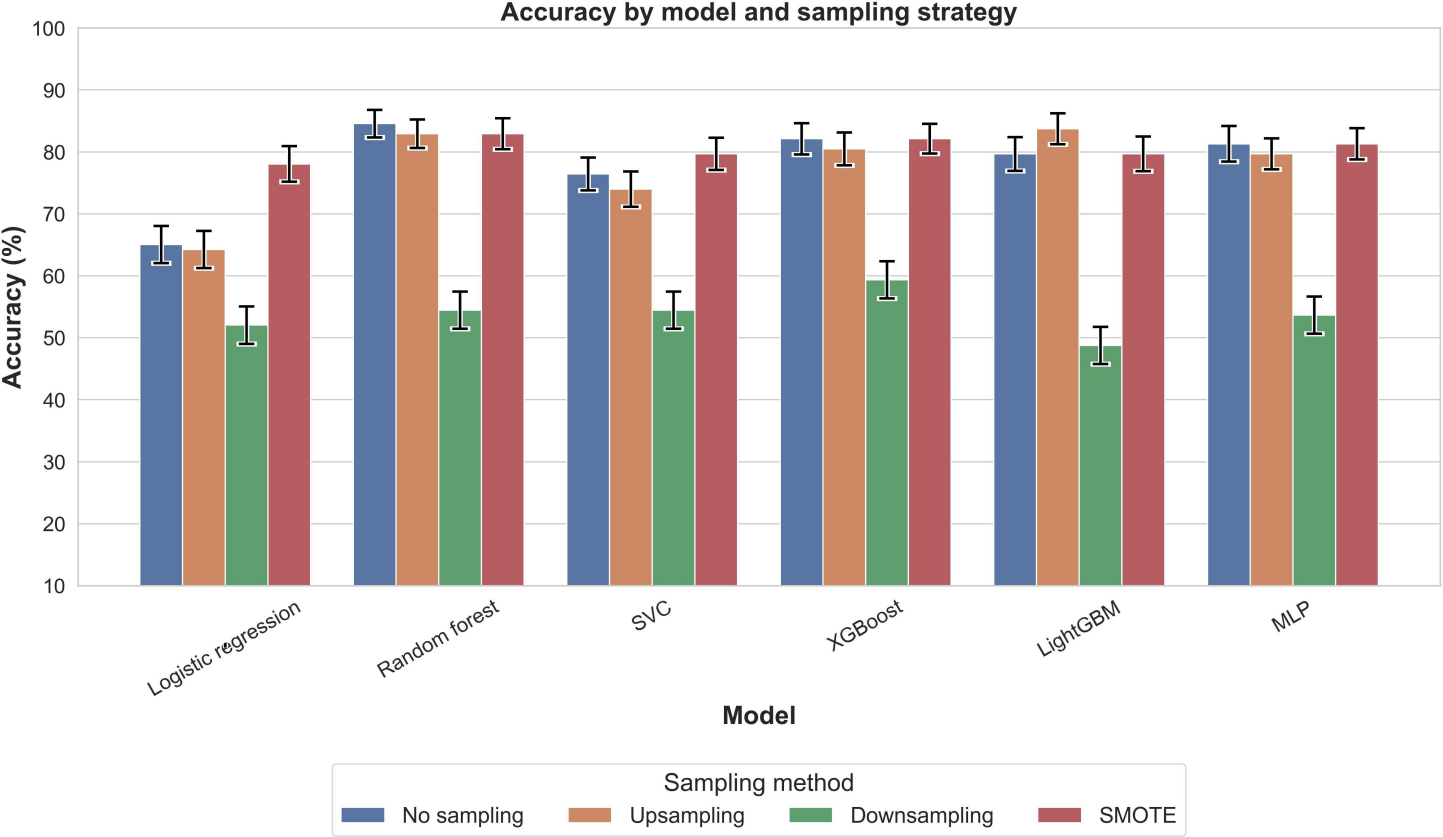
Comparison of accuracy across models and sampling methods for outcome night awakenings frequency. LightGBM: light gradient boosting machine; MLP: multilayer perceptron; SMOTE: synthetic minority oversampling technique; SVC: support vector classifier; XGBoost: extreme gradient boosting.

**Figure 8.**
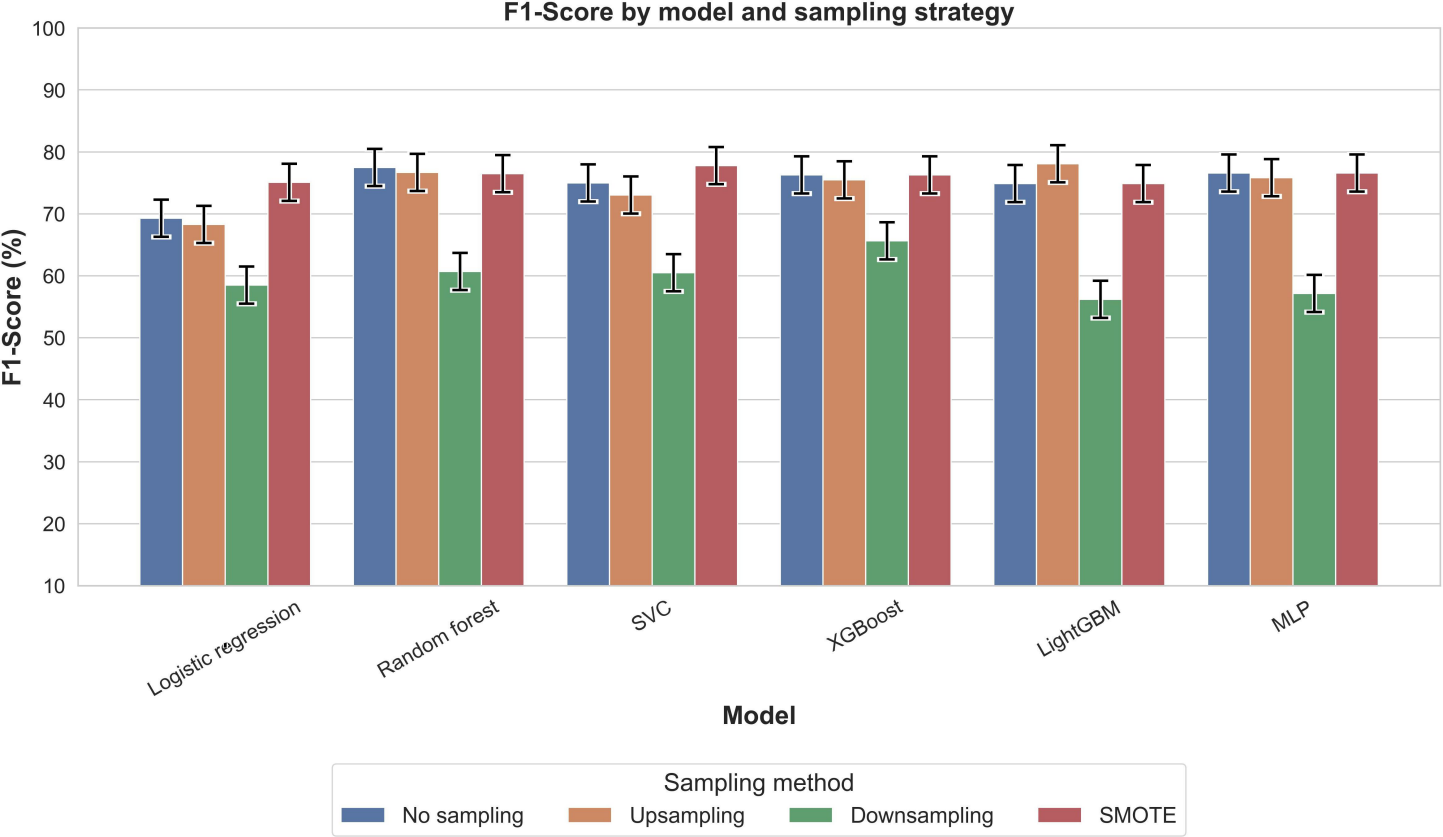
Comparison of *F*_1_-score across models and sampling methods for outcome night awakenings frequency. LightGBM: light gradient boosting machine; MLP: multilayer perceptron; SMOTE: synthetic minority oversampling technique; SVC: support vector classifier; XGBoost: extreme gradient boosting.

Figure 6 reports PR-AUC for predicting night awakenings frequency across models and sampling strategies. All models performed well, typically exceeding 0.83, with logistic regression highest (0.91) and MLP close behind (0.89). Figure 7 shows accuracy, with random forest without sampling highest (0.85) and MLP and XGBoost without sampling at 0.81; downsampling reduced accuracy for all models. *F*_1_-scores (Figure 8) mirrored accuracy, with MLP and XGBoost without sampling at 0.76 and random forest with SMOTE at 0.78. Models trained on downsampled data had the lowest *F*_1_-scores, underscoring the performance cost of sample reduction despite improved class balance.

#### Feature Importance Analysis

**Figure 9.**
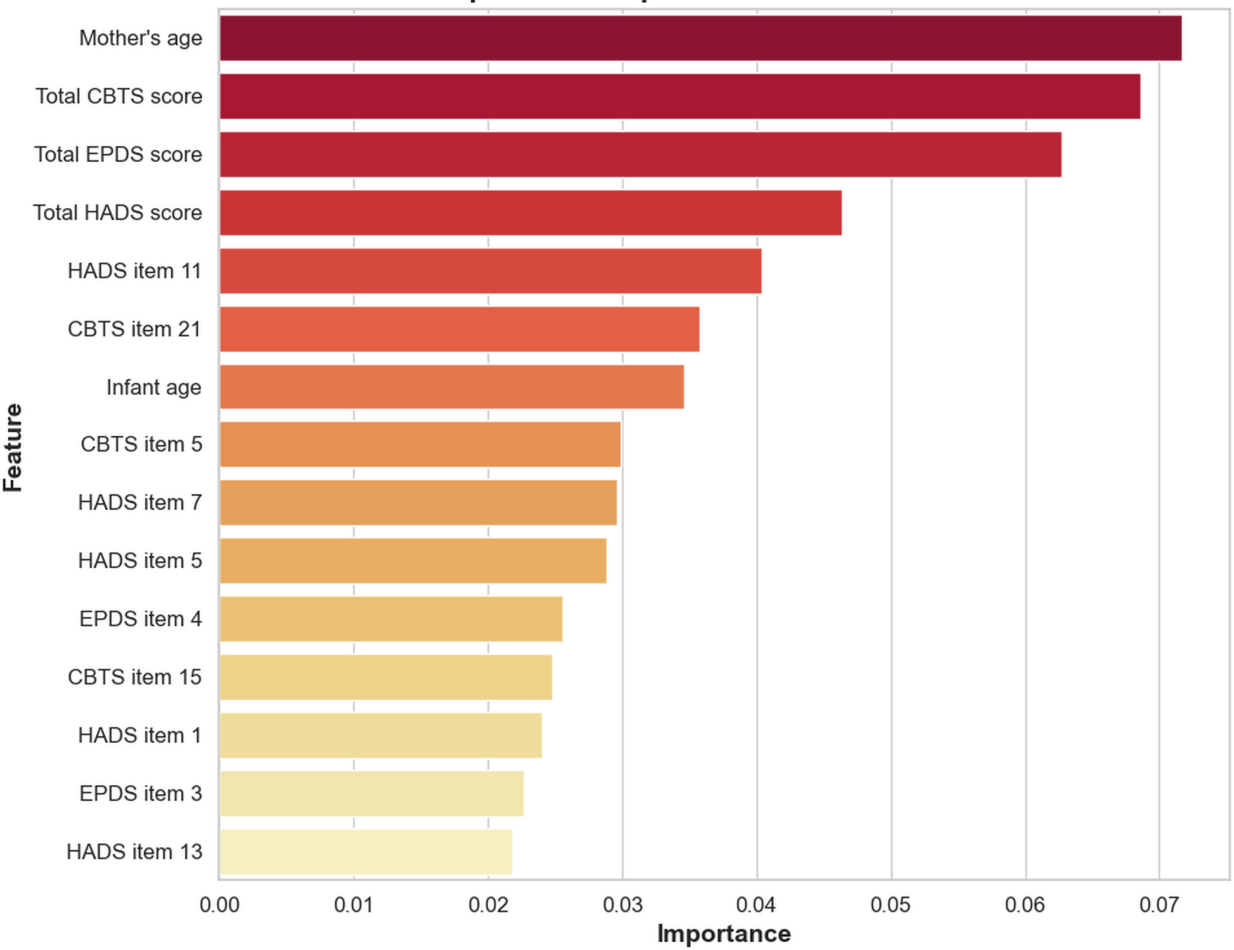
Feature importance analysis for outcome night awakenings frequency. EPDS: Edinburgh Postnatal Depression Scale; HADS: Hospital Anxiety and Depression Scale.

Feature importance analysis identified the most influential predictors of elevated night awakening frequency (Figure 9). As with nocturnal sleep duration, maternal age and total EPDS, HADS-A, and CBTS scores were top predictors. Individual items also contributed, notably HADS-A Item 11 (I feel restless and cannot seem to stay still) and CBTS Item 21 (Having difficulty concentrating). Infant age also emerged as a relevant predictor. An analogous SHAP summary plot for night awakenings (MultimediaAppendices 1^3^) confirms the prominence of MMH features and illustrates how variations in these scores and sociodemographic factors shift individual predictions toward higher or lower night-awakening risk.

## Discussion

### Key Findings

This study evaluated the utility of postpartum MMH measures in predicting infant sleep patterns using an ML approach. Notably, supervised ML models trained on standardized psychological screening instruments (EPDS, HADS-A, and CBTS), combined with basic demographic and maternal variables, demonstrated high predictive accuracy for both outcomes: insufficient nocturnal sleep duration and frequent night awakenings. These findings indicate the feasibility of using MMH symptoms to identify infants at risk for suboptimal sleep patterns. It also confirms the feasibility of integrating ML tools into postpartum care pathways to facilitate early risk identification.

### Predictors of Infant Sleep Patterns

Maternal age emerged as a top predictor for both outcomes, potentially reflecting links with parenting experience, physiological resilience, and contextual factors such as social support and caregiving efficacy. Prior work associating younger age with higher postpartum depression and poorer infant sleep [7,31] is consistent with its predictive strength in our models.

Total scores from the EPDS, HADS-A, and CBTS were among the most influential features in both models. Higher total EPDS scores indicate more severe postpartum depressive symptoms, which can impact maternal responsiveness and infant sleep regulation. Elevated anxiety levels (HADS-A) in mothers may lead to increased nighttime interactions, potentially disrupting infant sleep. Additionally, higher CBTS scores reflect greater CB-PTSD symptoms, which can affect maternal-infant bonding and sleep routines. These aggregate scores likely reflect the cumulative burden of postpartum psychological distress, which has been linked in prior research to disruptions in maternal caregiving behavior, nighttime responsiveness, and the emotional climate surrounding infant sleep routines [13,32,33].

Beyond aggregate symptom scores, several individual questionnaire items provided fine-grained insights. For example, EPDS Item 2 (“I have looked forward with enjoyment to things”)—a measure of anhedonia—was highly predictive of short nocturnal sleep duration. Lower scores on this item suggest anhedonia, a core symptom of depression, which may influence maternal engagement in establishing infant sleep routines. Similarly, CBTS Item 15 (“I felt distant or cut off from other people”) was ranked highly predictive of short nocturnal sleep duration, suggesting that maternal emotional withdrawal and social detachment, characteristic of childbirth-related trauma, may negatively impact the ability to establish secure and consistent nighttime routines.

In the models predicting frequent night awakenings, several additional features emerged as specific to this outcome. These included HADS-A Item 11 (“I feel restless and can’t seem to sit still”) and CBTS Item 21 (“I had difficulty concentrating”), both of which reflect maternal hyperarousal and cognitive dysregulation. These symptoms may manifest in heightened maternal vigilance or difficulty in promoting infant self-soothing, thereby contributing to fragmented infant sleep. Infant age also appeared as a differentiating predictor for this outcome, likely reflecting developmental maturation of sleep consolidation and age-dependent thresholds used in classifying night awakening frequency.

### Research and Clinical Implications

From a research perspective, this study illustrates the value of combining symptom-level data with advanced modeling approaches to move beyond correlational frameworks and toward predictive analytics in maternal–infant health. The identification of both composite scores and individual symptom items as key predictors offers a granular understanding of how distinct psychological dimensions—such as anhedonia, emotional detachment, and hyperarousal—may differentially impact infant sleep regulation. These findings advocate for future investigations that examine not only the additive burden of MMH symptoms, but also the specific affective and cognitive pathways through which maternal distress shapes caregiving practices and infant behavioral development. Because MMH and infant sleep outcomes were assessed at the same time point, our models characterize concurrent statistical associations rather than temporal or causal effects. In this context, we use the term “prediction” to denote out-of-sample statistical prediction within the cross-sectional dataset, not longitudinal forecasting. The original analysis of this dataset by Sandoz et al [12] examined the cross-sectional associations between MMH symptom profiles and infant sleep outcomes using traditional statistical methods. In contrast, the present study focuses exclusively on evaluating the predictive performance of supervised ML models that use these MMH measures to classify infant sleep outcomes. Longitudinal studies are particularly needed to clarify the temporal sequence between MMH symptom fluctuations and changes in infant sleep architecture. Moreover, item-level granularity opens avenues for psychometric refinement of postpartum screening instruments, enabling the development of targeted subscales that better predict specific infant outcomes.

Integrating wearable technologies (eg, smartwatches, sleep trackers, biosensors) could passively capture continuous physiological and behavioral data from mothers and infants, reducing reliance on retrospective self-report. When combined with symptom-level psychological data, these rich data streams may improve ML predictive accuracy, enable earlier detection of risk patterns, and support more responsive, personalized interventions.

From a clinical perspective, our findings are best viewed as proof of concept for generating individualized risk scores rather than as a ready-to-deploy screening tool. In practice, such risk scores could be integrated into routine postpartum or well-baby contacts to flag mother-infant dyads who may benefit from closer follow-up (eg, additional monitoring visits or phone check-ins), brief psychoeducation on infant sleep and maternal self-care, targeted support around bedtime routines and soothing strategies, or referral to perinatal mental health services for more structured interventions (such as brief CBT-based programs, parenting support groups, or trauma-focused care where indicated). The exact decision thresholds would need to be codesigned with clinicians and policymakers, balancing sensitivity (minimizing missed high-risk dyads) against specificity and available resources. Our analyses therefore focus on overall discrimination metrics (eg, PR-AUC, *F*_1_) rather than on a single “optimal” cut-off; future work should calibrate and validate context-specific thresholds and decision rules in real-world postpartum care pathways. In addition, findings from this study may inform the design of preventive intervention trials. For instance, trials could test whether tailoring interventions to specific symptom clusters (eg, anhedonia-focused therapies for mothers at risk of short infant sleep duration) yields superior outcomes. Finally, these results highlight the importance of interdisciplinary collaboration—integrating mental health, pediatrics, and data science—to advance personalized, responsive, and developmentally informed postpartum care that promotes long-term maternal and infant well-being.

### Limitations and Future Directions

Several limitations should be carefully considered when interpreting the findings of this study.

First, the data relied entirely on maternal self-report questionnaires, which introduces potential response and recall biases. Mothers experiencing psychological distress may perceive or report their infant’s sleep differently, potentially inflating associations between MMH symptoms and infant sleep disturbances due to shared method variance. Furthermore, infant sleep during the first year is influenced by a complex interplay of biological, environmental, and caregiving factors. The exclusive focus on MMH, without integrating other relevant variables such as infant temperament, feeding methods, family routines, or the home sleep environment, limits the comprehensiveness of the predictive models. Future studies should incorporate multimodal, multi-informant data sources, including reports from partners or caregivers and objective sleep measures such as actigraphy or polysomnography, alongside contextual and behavioral variables to more accurately capture the multifactorial nature of infant sleep regulation.

Second, the analysis was limited to 409 mother-infant dyads, all recruited from a single university hospital in Switzerland. This relatively modest sample size and geographically restricted setting may limit the generalizability of the findings to broader, more diverse populations. Sociocultural factors, health care systems, parental practices, and support structures can vary significantly across regions and may influence both MMH and infant sleep patterns. Future studies should validate these predictive models using larger, more heterogeneous samples across multiple countries and health care settings to ensure greater external validity and applicability of the results.

Third, the cross-sectional design limits causal inference. Although we examine associations between MMH symptoms and infant sleep, we cannot determine directionality or temporality. MMH may influence infant sleep, but the reverse is also plausible, with persistent sleep disturbances worsening maternal distress. Longitudinal studies are needed to disentangle these bidirectional effects and to capture trajectories of MMH and infant sleep over time.

### Conclusions

This study demonstrates the feasibility and utility of applying supervised ML models to postpartum MMH symptom measures, together with basic maternal–infant characteristics, to predict infant sleep outcomes—specifically nocturnal sleep duration and night awakening frequency—during the first year of life. The combination of high-performing models and consistent variable importance patterns suggests that both maternal psychological well-being (eg, depressive, anxiety, and CB-PTSD symptoms) and non–mental-health factors such as maternal and infant age are associated with infant sleep patterns in this sample. By integrating scalable mental health screening tools with predictive analytics, this approach holds promise for early identification of at-risk dyads and for informing targeted, preventive interventions that support both maternal and infant health outcomes.

## Supplementary material

10.2196/78937 Multimedia Appendix 1 Data Dictionary.

10.2196/78937 Multimedia Appendix 2 Shapley additive explanations (SHAP) summary plot for nocturnal sleep disturbance.

10.2196/78937 Multimedia Appendix 3 Shapley additive explanations (SHAP) summary plot for night awakening.

## Abbreviations

BISQ: Brief Infant Sleep Questionnaire
CB-PTSD: childbirth-related post-traumatic stress disorder
CBTS: City Birth Trauma Scale
ECG: electrocardiogram
EPDS: Edinburgh Postnatal Depression Scale
HADS-A: Hospital Anxiety and Depression Scale-Anxiety Subscale
LGBM: light gradient boosting machine
ML: machine learning
MLP: multilayer perceptron
MMH: maternal mental health
PR-AUC: precision-recall area under the curve
SHAP: Shapley additive explanations
SMOTE: synthetic minority oversampling technique
XGBoost: extreme gradient boosting
