# Supplementary material for: Predicting Infant Sleep Patterns From Postpartum Maternal Mental Health Measures: Machine Learning Approach
Source: JMIR Pediatr Parent. 2026 Feb 3;9:e78937. doi: 10.2196/78937 (PMC12867479; doi:10.2196/78937)
Supplement: Multimedia Appendix 1 [file pediatrics-v9-e78937-s001.docx]

**Appendix 1: Data Dictionary**

| **Variable name** | **Variable description** | **Additional information** |
| --- | --- | --- |
|  | | |
| **Demographic and Maternal Variables** | | |
| Mother's age | Item determining participant's age in years | min = 19, max = 47 |
| Mother’s education | Item determining participant's educational level | 1 = no education ; 2 = compulsory school ; 3 = post-compulsory education ; 4 = university of Applied Science or University Technology Degree ; 5 = university. |
| Gestational age | Item determining participant's weeks of gestation | min = 26, max = 43 |
| Pregnancy type | Item determining the type of pregnancy | 1 = single pregnancy; 2 = twin pregnancy (only 1st baby info is used in the analysis) |
| Infant age | Item determining the age category of the infant | 1 = ≥3 months to <6 months; 2 = ≥6 months to <9 months; 3 = ≥9 months to <12 months |
| Infant sex | Item determining the sex of the infant | 1 = girl; 2 = boy |
|  | | |
| **Edinburgh Postnatal Depression Scale** | | |
| EPDS_1 | Item 1 of the Edinburgh Postnatal Depression Scale | Q: I have been able to laugh and see the funny side of things. |
| EPDS_2 | Item 2 of the Edinburgh Postnatal Depression Scale | Q: I have felt confident and joyful when thinking about the future. |
| EPDS_3 | Item 3 of the Edinburgh Postnatal Depression Scale | Q: I have blamed myself unnecessarily when things went wrong. |
| EPDS_4 | Item 4 of the Edinburgh Postnatal Depression Scale | Q: I have felt anxious or worried for no good reason. |
| EPDS_5 | Item 5 of the Edinburgh Postnatal Depression Scale | Q: I have felt scared or panicked for no real reason. |
| EPDS_6 | Item 6 of the Edinburgh Postnatal Depression Scale | Q: I have felt overwhelmed by events. |
| EPDS_7 | Item 7 of the Edinburgh Postnatal Depression Scale | Q: I have felt so unhappy that I had trouble sleeping. |
| EPDS_8 | Item 8 of the Edinburgh Postnatal Depression Scale | Q: I have felt sad or not very happy. |
| EPDS_9 | Item 9 of the Edinburgh Postnatal Depression Scale | Q: I have felt so unhappy that I have cried. |
| EPDS_10 | Item 10 of the Edinburgh Postnatal Depression Scale | Q: I have had thoughts of harming myself. |
|  | | |
| **Hospital Anxiety and Depression Scale (anxiety subscale)** | | |
| HADS_1 | Item of the Hospital Anxiety and Depression Scale (anxiety subscale) | Q: I feel tense or nervous. |
| HADS_3 | Item of the Hospital Anxiety and Depression Scale (anxiety subscale) | Q: I have a feeling of fear as if something terrible is going to happen. |
| HADS_5 | Item of the Hospital Anxiety and Depression Scale (anxiety subscale) | Q: I worry a lot. |
| HADS_7 | Item of the Hospital Anxiety and Depression Scale (anxiety subscale) | Q: I can sit quietly and feel relaxed. |
| HADS_9 | Item of the Hospital Anxiety and Depression Scale (anxiety subscale) | Q: I experience feelings of fear and have a knot in my stomach. |
| HADS_11 | Item of the Hospital Anxiety and Depression Scale (anxiety subscale) | Q: I feel restless and can’t seem to stay still. |
| HADS_13 | Item of the Hospital Anxiety and Depression Scale (anxiety subscale) | Q: I have sudden feelings of panic. |
|  | | |
| **Maternal City Birth Trauma Scale** | | |
| CBTS_M_3 | Item 3 of the maternal City Birth Trauma Scale | Q: Repeated and involuntary memories of the birth (or parts of the birth) that you cannot control. |
| CBTS_M_4 | Item 4 of the maternal City Birth Trauma Scale | Q: Bad dreams or nightmares about the birth, or related to the birth. |
| CBTS_M_5 | Item 5 of the maternal City Birth Trauma Scale | Q: Flashbacks of the birth and/or reliving the experience. |
| CBTS_M_6 | Item 6 of the maternal City Birth Trauma Scale | Q: Feeling distressed when something reminds you of the birth. |
| CBTS_M_7 | Item 7 of the maternal City Birth Trauma Scale | Q: Feeling tense or anxious when something reminds you of the birth. |
| CBTS_M_8 | Item 8 of the maternal City Birth Trauma Scale | Q: Trying to avoid thinking about the birth. |
| CBTS_M_9 | Item 9 of the maternal City Birth Trauma Scale | Q: Trying to avoid things that remind you of the birth (e.g., people, places, TV shows, etc.). |
| CBTS_M_10 | Item 10 of the maternal City Birth Trauma Scale | Q: Unable to remember details of the birth. |
| CBTS_M_11 | Item 11 of the maternal City Birth Trauma Scale | Q: Blaming yourself or others for what happened during the birth. |
| CBTS_M_12 | Item 12 of the maternal City Birth Trauma Scale | Q: Having intense negative emotions about the birth (e.g., fear, anger, shame). |
| CBTS_M_13 | Item 13 of the maternal City Birth Trauma Scale | Q: Having negative beliefs about yourself or fearing that something terrible will happen. |
| CBTS_M_14 | Item 14 of the maternal City Birth Trauma Scale | Q: Losing interest in activities that were previously important to you. |
| CBTS_M_15 | Item 15 of the maternal City Birth Trauma Scale | Q: Feeling detached from others. |
| CBTS_M_16 | Item 16 of the maternal City Birth Trauma Scale | Q: Unable to feel positive emotions (e.g., joy, excitement). |
| CBTS_M_17 | Item 17 of the maternal City Birth Trauma Scale | Q: Feeling irritable or aggressive. |
| CBTS_M_18 | Item 18 of the maternal City Birth Trauma Scale | Q: Having self-destructive feelings or engaging in reckless behavior. |
| CBTS_M_19 | Item 19 of the maternal City Birth Trauma Scale | Q: Feeling tense and on edge. |
| CBTS_M_20 | Item 20 of the maternal City Birth Trauma Scale | Q: Feeling restless or easily startled. |
| CBTS_M_21 | Item 21 of the maternal City Birth Trauma Scale | Q: Having difficulty concentrating. |
| CBTS_M_22 | Item 22 of the maternal City Birth Trauma Scale | Q: Having trouble sleeping due to reasons unrelated to the baby’s sleep pattern. |
|  | | |
| **Brief Infant Sleep Questionnaire (BISQ)** | | |
| Sleep night duration | Item of the Brief Infant Sleep Questionnaire assessing nocturnal sleep duration (between 7pm and 7 am) | Responses coded as hh:mm. |
| Night awakening number | Item of the Brief Infant Sleep Questionnaire assessing the number of night waking | Average number of night awakenings. |
