# Supplementary material for: Predicting Infant Sleep Patterns From Postpartum Maternal Mental Health Measures: Machine Learning Approach
Source: JMIR Pediatr Parent. 2026 Feb 3;9:e78937. doi: 10.2196/78937 (PMC12867479; doi:10.2196/78937)
Supplement: Multimedia Appendix 3 [file pediatrics-v9-e78937-s003.docx]

**Appendix 3:** **SHAP summary plot for night awakening.**


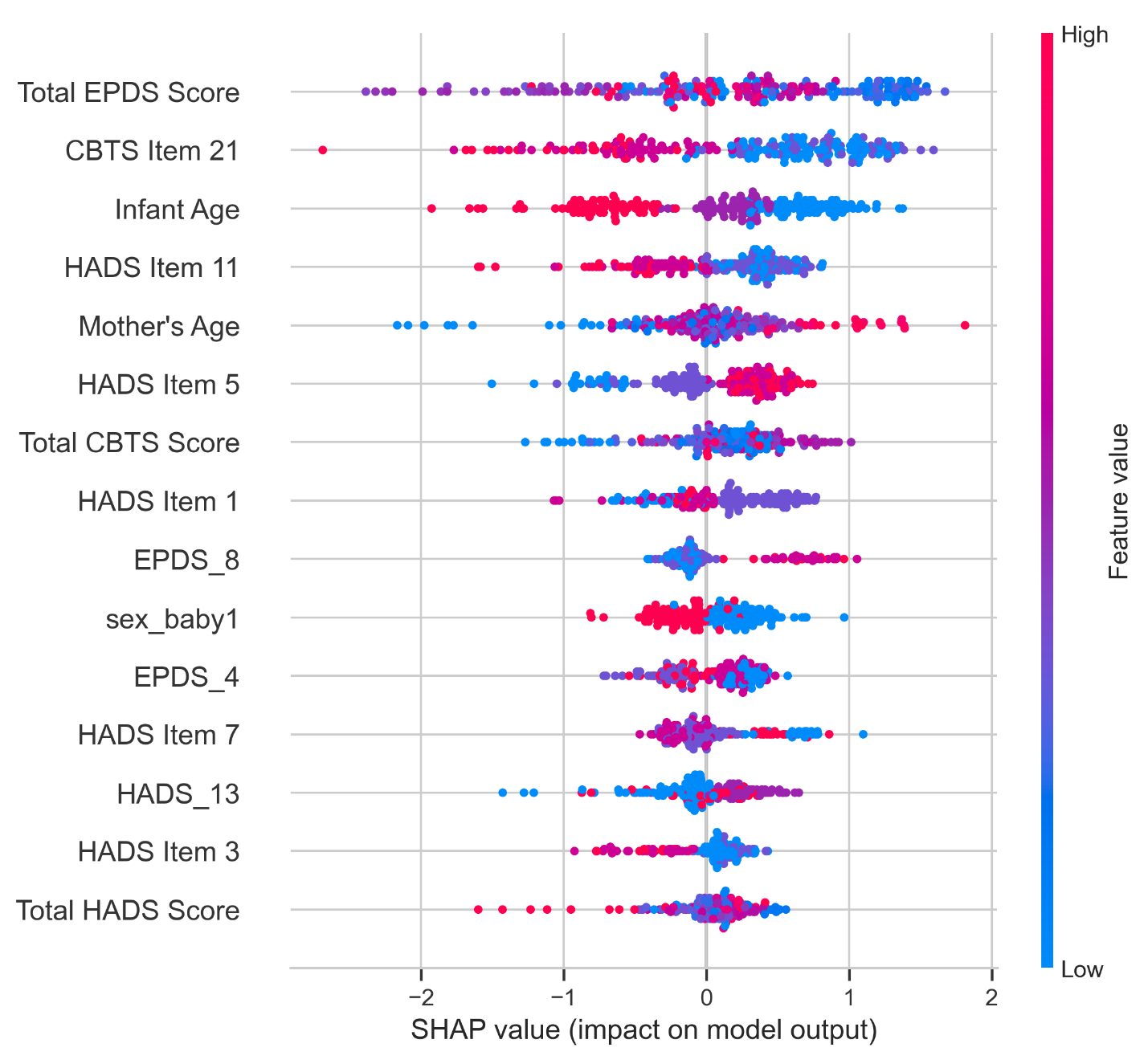


**Figure S2.** SHAP summary plot for the machine-learning model predicting night awakening. Each dot represents one participant, with color indicating the feature value (blue = low, pink = high) and position on the x-axis showing the SHAP value, i.e., the direction and magnitude of that feature’s contribution to the predicted probability of night awakening. Features are ordered from top to bottom by their overall importance (mean absolute SHAP value)
